# Effect of Sudden Deprivation of Sensory Inputs From Periodontium on Mastication

**DOI:** 10.3389/fnins.2019.01316

**Published:** 2019-12-10

**Authors:** Anastasios Grigoriadis, Abhishek Kumar, Magnus K. Åberg, Mats Trulsson

**Affiliations:** ^1^Section of Oral Rehabilitation, Division of Oral Diagnostics and Rehabilitation, Department of Dental Medicine, Karolinska Institutet, Huddinge, Sweden; ^2^Scandinavian Center for Orofacial Neurosciences, Huddinge, Sweden; ^3^Department of Environmental Science and Analytical Chemistry, Stockholm University, Stockholm, Sweden

**Keywords:** temporal profile, jaw muscle activity, periodontal mechanoreceptors, viscoelastic hard food, muscle activation, jaw kinematics

## Abstract

**Objective:**

To investigate the effect of sudden deprivation of sensory inputs from the periodontium on jaw kinematics and time-varying activation profile of the masseter muscle.

**Methods:**

Fourteen (age range: 22–26 years; four men) healthy and natural dentate volunteers participated in a single experimental session. During the experiment, the participants were asked to eat six hard visco-elastic test food models, three each before and after an anesthetic intervention. The movements of the jaw in three dimensions and electromyographic (EMG) activity of the masseter muscle on the chewing side were recorded.

**Results:**

The results of the study showed no significant differences in the number of chewing cycles (*P* = 0.233) and the duration of chewing sequence (*P* = 0.198) due to sudden deprivation of sensory inputs from the periodontium. However, there was a significant increase in the jaw opening velocity (*P* = 0.030) and a significant increase in the duration of occlusal phase (*P* = 0.004) during the anesthetized condition. The EMG activity of the jaw closing phase was significantly higher during the control condition [116.5 arbitrary units (AU)] than anesthetized condition (93.9 AU). The temporal profile of the masseter muscle showed a biphasic increase in the excitatory muscle drive in the control condition but this increase was virtually absent during the anesthetized condition.

**Conclusion:**

Sudden deprivation of sensory inputs from the periodontium affects the jaw kinematics and jaw muscle activity, with a clear difference in the time-varying activation profile of the masseter muscle. The activation profile of the masseter muscle shows that periodontal mechanoreceptors contribute to approximately 20% of the EMG activity during the jaw closing phase.

## Introduction

The rhythmic masticatory movements of the jaws help in the physical breakdown of food morsels into smaller particles and form a soft bolus suitable for swallowing. The central pattern generators located in the brain stem are responsible for generating the basic rhythmic jaw movements (Dellow and Lund, 1971; Lund, 1991; Lund et al., 1998). The afferent sensory information from in and around the oral cavity modify and fine-tune the jaw movements. One important class of somatosensory receptors is located in the collagen fibers within the periodontal ligament space of the tooth root. These primary afferent receptors are called periodontal mechanoreceptors (PMRs). The PMRs are efficient in encoding specific information for modulating the jaw motor neuron activity responsible for regulation of forces and jaw movements during chewing (Lund, 1991; Trulsson, 2006). Specifically, it is suggested that the sensory information from the PMRs are used by the central nervous system to optimize food positioning between the teeth and regulate the force levels and force vectors involved in biting. Further, the forces required in regulating the masticatory movements can also be influenced by the motor cortex (Sessle, 2006; Sessle et al., 2007, 2013; Avivi-Arber et al., 2011). Thus, mastication is a semiautomatic, subconscious activity that can be brought to conscious control according to the specifics of task demand (Westberg and Kolta, 2011).

The jaw muscle activity adapts to the changing properties/hardness of the food during the masticatory sequence. The jaw muscle activity is generally higher while chewing harder food than chewing softer food (Peyron et al., 2002; Grigoriadis et al., 2011, 2014; Iguchi et al., 2015; Grigoriadis and Trulsson, 2018). However, people lacking PMRs as in case of patients with implant supported bridges show an impaired adaptation to food hardness (Grigoriadis et al., 2011). While both “implant patient” and the “naturally dentate” groups show similar behavior in chewing soft food morsels, the implant patient group particularly demonstrate signs of impairment while chewing harder food (Grigoriadis et al., 2011). It has been previously shown that the adaptation to the physical characteristics of the food (including its rheological properties) is caused by changes in the muscle commands that alter jaw kinematics and chewing forces (Ottenhoff et al., 1992, 1993; Grigoriadis et al., 2011). These studies have shown that a major fraction of the observed jaw muscle activity also referred to as the “additional muscle activity” is used to overcome the resistance offered by the food hardness during the act of chewing (Ottenhoff et al., 1992, 1993). While a smaller fraction of this muscle activity is also utilized to move the jaw. However, the muscle build up for the jaw movements can partially also occur in anticipation to the tooth food contact (Westberg and Kolta, 2011).

Studies on anesthetized rabbits indicate that the component of “additional muscle activity” that precedes the early tooth–food contact during chewing is unaffected by blocking the PMRs. Likewise, other animal studies suggest that signals from muscle spindles are most important during the early phases of force generation, whereas inputs from both muscle spindles and PMRs are important during the later phases (Lavigne et al., 1987; Morimoto et al., 1989). Previously, we have shown impaired force control and impaired spatial regulation in healthy young adults after anesthesia of the teeth during biting tasks (Trulsson and Johansson, 1996; Grigoriadis et al., 2017; Kumar et al., 2017). We have previously also described the chewing sequence, chewing cycle, and time-varying activation profile of the masseter muscle and how food hardness affects the profile during natural chewing in healthy participants (Grigoriadis et al., 2014). Subsequently, it was shown that unlike natural dentate participants who demonstrate increased ability to adapt jaw muscle activity according to the food hardness, the implant prosthesis patients fail to adapt/increase the jaw muscle activity (Grigoriadis et al., 2011). However, the degree of contribution of the PMRs in the regulation of jaw muscle activity during chewing has not been studied. Therefore, in the current study we investigated the effect of sudden deprivation of sensory inputs from the periodontium on jaw kinematics and time-varying activation profile of the masseter muscle. We hypothesized that sudden deprivation of sensory inputs would alter the jaw movement kinetics and jaw muscle activity along with changes in the time-varying activation profile of the masseter muscles similar to the findings from implant patients in the previous studies (Grigoriadis et al., 2011; Grigoriadis and Trulsson, 2018).

## Materials and Methods

### Study Participants

The study included 14 (age range: 22–26 years; four men) healthy and natural dentate participants with at least 28 permanent teeth. At the time of the experiment none of the participants reported, nor indicated, any problems or dysfunctions related to biting or chewing behavior. The Regional Ethical Review Board, Stockholm, Sweden, approved the study and all participants gave written informed consent in accordance with the Declaration of Helsinki II, prior to the start of the experiment.

### Experimental Protocol

The participants voluntarily participated in a single experimental session. During the experiment the participants were asked to chew and swallow a hard visco-elastic test food models three times each (total six trials) before and after an anesthetic intervention. The participants were also asked about their preferred chewing side and were instructed to chew only on the preferred chewing side throughout the experiment. The recipe for the preparation of the visco-elastic model foods has been previously described in detail, see Grigoriadis et al. (2011) for more information. The test food model was cylindrical (20 mm × 10 mm) in shape with a hardness of about 129 ± 21 kPa. Before the start of each trial, the experimenter placed the test food morsel on the extended tongue of the participant. The participants were asked to hold the test food between the tongue and the palate with the mouth closed and their teeth in maximum intercuspation for about 2–4 s. Further, the experimenter signaled the participants to start chewing and place the teeth back again in intercuspation once the morsel was swallowed. Between trials, the participants were free to drink, rest, speak, and rinse the mouth if they desired to. The participants were not given any specific information regarding the objective of the study prior to the start of the experiment.

### Anesthetic Intervention

A computer-assisted system for local anesthesia (The Wand^®^, Milestone Scientific, Livingston, NJ, United States) was used to anesthetize the teeth on the preferred chewing side of both the jaws. Anesthesia was achieved by local infiltration and periodontal injections of approximately 6 × 1.8 ml anesthetic solution (Citanest^®^, Dentsply Sirona, Sweden). The participants were asked to confirm the subjective symptoms associated with local anesthesia and these symptoms were objectively evaluated by lack of response to light touch and pressure to the anesthetized teeth.

### Recording Jaw Movements and Muscle Activity

**FIGURE 1:**
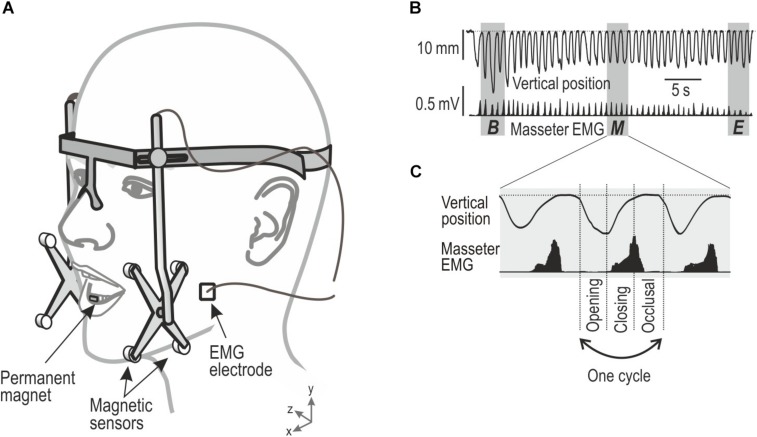
Showing experimental setup of jaw movements and electromyographic (EMG) activity and description of chewing sequence and chewing cycles obtained during chewing. **(A)** Custom-built device for monitoring the mandibular movements in three dimensions, EMG activity from the masseter muscle was recorded by bipolar surface electrodes. **(B)** Vertical position of the mandible and EMG activity (root-mean-square processed) during a masticatory sequence. Gray area indicates segments (three cycles) of the masticatory sequence representing its beginning (B), middle (M), and end (E) of the chewing sequence. **(C)** Each cycle were divided in three phases, Opening, Closing, and Occlusal.

The apparatus and the general description of the armamentariums used in the current experiment have been described in detail in our earlier publications; see Grigoriadis et al. (2011, 2014), Svensson et al. (2013), Kumar A. et al. (2015), and Grigoriadis and Trulsson (2018). Briefly, movements of the lower jaw in reference to the upper jaw were measured using a custom build three dimensional, jaw movement tracking equipment (Department of Integrative Medical Biology, Umeå University, Umeå, Sweden). The labial surface of mandibular central incisors was etched and a small magnet (10 × 5 × 5 mm) was attached with dental composites. A lightweight frame equipped with eight magnetic sensors (four on each side) that tracked the position of the magnet in all three dimensions (accuracy: 0.1 mm; bandwidth: 0–100 Hz) was attached to the head of the participant in a spectacle-like frame (Figure 1A). The frame was further secured with adjustable straps. The electromyographic (EMG) activity of the masseter muscle was recorded with customized, bipolar, surface electrodes (Department of Integrative Medical Biology, Umeå University, Umeå, Sweden). The skin over the masseter muscle was cleansed thoroughly with alcohol and electrodes (2 mm in diameter and 12 mm apart) were carefully placed on the muscle after palpation. The data acquired was stored using the SC/ZOOM microcomputer-based data acquisition and analyzed with customized software (SC/ZOOM, v. 3.1.02, Physiology Section, IMB, Umeå University, Umeå, Sweden). Further, the data acquired was transferred to Matlab (Version R2010b, The MathWorks, Inc.) for analysis.

### Data Analysis

The outcome variables and the areas of interest were similar to our previous studies (Grigoriadis et al., 2014; Grigoriadis and Trulsson, 2018). The jaw movement kinematics were established to study the number of chewing cycles and the duration of the chewing sequence during each trial. The vertical and lateral amplitude of the jaw movements during a single chewing cycle along with the peak velocity of the jaw during jaw opening and jaw closing were also measured in the vertical and lateral dimension. The analysis was focused on three segments that represented the beginning, middle, and end of each chewing sequence (Figure 1B). Each segment was represented by the mean of three consecutive cycles in the beginning, middle, and the end of the chewing sequence. The first and the last cycle of the sequence were excluded due to great intra-individual variability across trials. Thus, the first and the last segment represented about second to fourth cycles during the onset and offset of the chewing sequence, respectively. The segment representing the middle of the sequence included the three cycles located in its center.

Each chewing cycle consisted of a jaw-opening phase, followed by a jaw closing phase and an occlusal phase (Figure 1C). The start of the jaw-opening phase was characterized by the opening of the jaw from the occlusal state by 1 mm. The occlusal state was characterized for each participant as the minimum jaw opening recorded during each trial, including the periods when the participants’ teeth were in maximum intercuspation. The jaw opening phase ended at peak jaw opening and was followed by the jaw closing phase which subsequently ended when the jaw reached the same vertical jaw position as when the jaw opening phase begun (Grigoriadis et al., 2014). Finally, the occlusal phase, started at the end of the closing phase, and ended at the beginning of the jaw-opening phase of the subsequent chewing cycle (Grigoriadis et al., 2014).

The EMG signals were sampled at 3.2 kHz and thereafter the root mean square (RMS) of the EMG signals was processed over a moving time window corresponding to 100 samples (31 ms). The RMS-processed signals were integrated during each phase of each chewing cycle giving each phase a measure corresponding to the area under the RMS−processed EMG signal. The total EMG activity for each chewing cycle was also computed as the sum of the integrated electromyograms for each of the three phases (Grigoriadis et al., 2011). The EMG data obtained from the mean EMG activity averaged across all chewing cycles were normalized to facilitate cross comparison of the EMG activity across participants. Specifically, the time-varying RMS processed EMG signals from each participant and each muscle was divided by the mean value of the EMG activity recorded from the muscle during all chewing cycles performed by the participant. This normalization allowed evaluation of relative effects of segment of the masticatory sequence on the time-varying activity in each of the four muscles recorded. To preserve phase information while combining data from different chewing cycles, the time base was normalized by scaling each phase of each cycle to the mean duration of that phase (Grigoriadis et al., 2011).

### Statistical Analysis

The data were checked for the assumptions of normal distribution with Shapiro–Wilk test and histogram plots. The data pertaining to the number of chewing cycles, duration of chewing cycle, jaw opening velocity, occlusal, and jaw-opening and jaw-closing duration did not appear to be normally distributed hence, non-parametric Wilcoxon sign-ranked tests were applied to test the differences between the conditions. All normally distributed data, i.e., frequency/rhythm of chewing, vertical and lateral jaw movements, and jaw closing velocity were analyzed with Student’s *t*-tests. The normally distributed data for the EMG activity of the masseter muscle were analyzed with two-way analysis of variance model (ANOVA) with repeated measures. The factors in ANOVA were conditions (two levels; control and anesthetized) and segments (three levels; beginning, middle, and end). *Post hoc* comparisons were done with Tukey’s HSD test. A *P*-value of <0.05 was decided to be significant. The percent changes in EMG activation between the control and anesthetized conditions during the jaw closing phase were calculated as (EMG activity during the jaw closing phase in the control condition − EMG activity during the jaw closing phase in the anesthetized condition)/EMG activity during the jaw closing phase in the control condition × 100.

## Results

The participants were able to perform the chewing task in a reliable manner under both the conditions, as instructed. All the participants confirmed the subjective symptoms related to local anesthesia after the anesthetic intervention. We have previously reported the quantitative parameters of jaw-movements, integrated EMG activity during the chewing cycles, and the adaptation of jaw muscle activity to food hardness in these healthy adults (Grigoriadis et al., 2014). In the current study, we will focus on the effect of anesthetic intervention on these parameters and compare them with control condition from the previous study (Grigoriadis et al., 2014). Table 1 presents the mean and standard deviations of all the outcome variables related to chewing sequence and jaw kinematics during both conditions.

**TABLE 1:** Showing mean and standard deviations of all the outcome variables related to chewing sequence and jaw kinematics.

		**Control**			**Anesthesia**		
Chewing sequence	1. Number of cycles	26.9 ± 13.8			27.7 ± 15.2		
	2. Duration of sequence (s)	20.1 ± 10.4			21.0 ± 10.9		
	3. Rhythm (Hz)	1.4 ± 0.3			1.4 ± 0.3		

		**Beginning**	**Middle**	**End**	**Beginning**	**Middle**	**End**

Jaw kinematics	4. Vertical amplitude (mm)	17.62.9	14.42.3	13.02.1	16.92.6	14.82.0	13.32.2
	5. Lateral amplitude (mm)	8.21.4	6.71.2	6.31.5	7.41.3	7.01.2	6.21.3
	6. Jaw opening velocity (mm/s)	86.128.2	78.822.4	72.718.7	93.431.2	87.923.7	76.020.5
	7. Jaw closing velocity (mm/s)	97.329.1	84.621.6	73.014.0	88.922.2	87.421.7	73.417.3
	8. Occlusal duration (s)	0.30.1	0.30.1	0.30.1	0.40.1	0.30.1	0.40.1
	9. Opening duration (s)	0.30.1	0.20.1	0.20.1	0.20.1	0.20.1	0.20.1
	10. Closing duration (s)	0.30.1	0.20.03	0.20.03	0.30.1	0.20.04	0.20.04

### Chewing Sequence

There were no significant differences in the number of chewing cycles (*P* = 0.233), the duration of chewing sequence (*P* = 0.198), and the subsequent frequency/rhythm of chewing (*P* = 0.424) between the anesthesia and control conditions.

### Jaw Kinematics

There were no significant differences in the vertical and lateral jaw amplitudes between the anesthesia and control conditions at the beginning (*P* = 0.196 and *P* = 0.053, respectively), middle (*P* = 0.352, and *P* = 0.379, respectively), and end (*P* = 0.486, and *P* = 0.379) of the chewing sequence. However, there was a significant increase in the jaw opening velocity at the middle (*P* = 0.030) of the chewing sequence during the anesthetized condition, and a significant increase in the duration of occlusal phase (*P* = 0.004) but a significant decrease in the jaw opening phase (*P* = 0.009) during the anesthetized condition than the control condition, at the beginning of the chewing sequence.

### EMG Activity

The EMG activity of the masseter muscle on the chewing side during the control and anesthetized conditions was evaluated. The results showed an overall significant effect of condition with EMG activity lower in the anesthetized condition than the control condition (main effect; *F* = 5.39, *P* = 0.037). Further, there was a significant decrease in the EMG activity in the middle and end in comparison to the beginning of the chewing sequence (main effect; *F* = 33.82, *P* < 0.001). However, there was no significant interaction between the conditions and the segments (*F* = 0.83, *P* = 0.447).

### Temporal Profile of the EMG Activity

**FIGURE 2:**
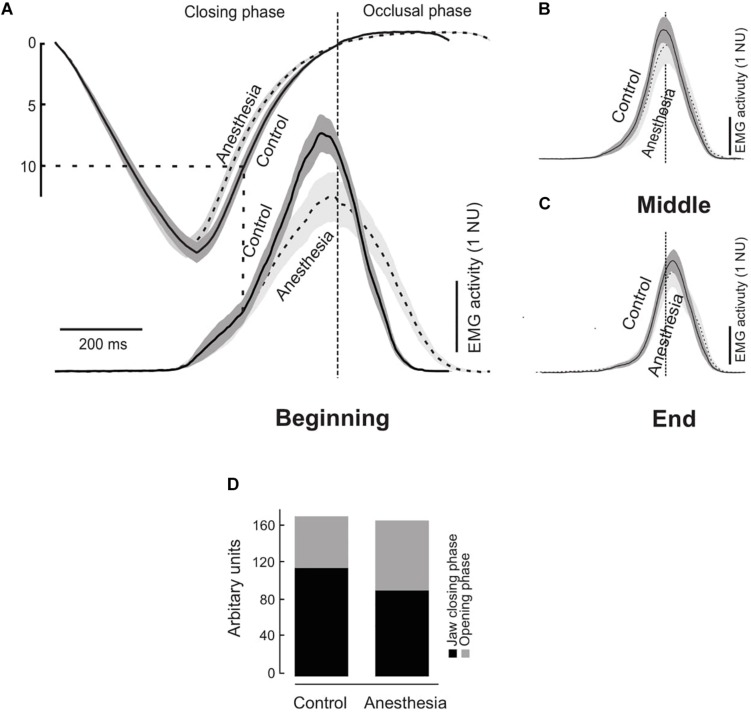
**(A)** Time normalized masseter muscle electromyographic (EMG) activity in normalized units (NU) during the control (solid curves) and anesthetized condition (dashed curves) during a chewing cycle at the beginning **(A)**, middle **(B)**, and end **(C)** of the chewing sequence. Gray areas indicate standard error of mean (*N* = 14) and the data have been aligned temporally at the start of the occlusal phase (time = 0). Gray dashed lines indicate the tooth food contact. Graph **(D)** shows components of the temporal profile curve expressed in arbitrary units for the jaw closing and occlusal phases during the control and anesthesia conditions.

The time dependent changes in the EMG activation are captured in the temporal profile of the EMG activity (Figures 2A–C). The temporal profile in the beginning of the chewing sequence during both the conditions showed a similar “bell-shaped” curve. The temporal profile at the beginning of the chewing sequence was characterized by an initial slow and later steep increase in the EMG activity during the jaw-closing phase and a clear peak followed by a declining EMG activity during the occlusal phase (Figure 2A). Both the curves appeared to be similar yet there were some noteworthy and distinct differences, which are discussed below.

A detailed analysis of the components of the temporal profile showed significantly lower peak of the EMG activity (highest point on the temporal profile of the EMG activity) expressed in arbitrary units (AU) during the anesthetized condition than the control condition (*P* = 0.003) (Figure 2A). Further, in the control condition the EMG activity during the jaw-closing phase (116.5 AU) was significantly higher than the occlusal phase (55.9 AU) (*P* < 0.001) (Figure 2D). However, there was no such difference in the anesthetized condition during jaw closing and occlusal phase (93.9 and 76.0 AU, respectively) (*P* = 0.198) (Figure 2D). The percent changes in EMG activation during the jaw closing phase were assessed by calculating the relative differences in the EMG activation between both the conditions. It was incidental that the EMG activity of the jaw-closing phase was approximately 20% lower during the anesthetized condition than the control (*P* = 0.009) but there was no such difference during the occlusal phase (*P* = 0.056).

The temporal profile activation of the masseter muscle on the chewing side during the beginning, middle, and end of the chewing sequence has been illustrated in Figures 2A–C, respectively. As reported earlier (Grigoriadis et al., 2014), the temporal profile on visual inspection showed a biphasic excitatory drive during the jaw closing phase in the control condition during the beginning of the chewing sequence. This biphasic excitatory drive clearly demonstrated an “early” and a “late” component during a single chewing cycle. The point of transition between the early and late component of the excitatory muscle drive occurred after the maximum jaw opening roughly corresponding to the size of the food morsel (∼11 mm). This transition is an indication of the time during the initial tooth food contact. However, the biphasic muscle drive was virtually absent during the anesthetized condition in the beginning of the masticatory sequence. Note that the cue to visually identify the biphasic phase is the exact time point when the tooth comes in contact with the food. During the initial stages of the chewing cycle the food dimensions are intact and food size is known (10 mm approximately in current experiment). However, since the food is cut and divided into several smaller pieces it is difficult to estimate the size of the food morsel and subsequently identify the occurrence of biphasic phases later in the chewing sequence. Hence, in the current study we have described the temporal profile of the jaw muscle activation only during the beginning of the chewing sequence.

## Discussion

We have previously shown altered motor function between naturally dentate controls and dental prosthesis patients during various biting (Trulsson and Gunne, 1998; Svensson et al., 2013) and chewing tasks (Grigoriadis et al., 2011, 2015). It has been suggested that the impaired masticatory performance in prosthodontic patients is primarily due to the lack of sensory information from the PMRs and neuromuscular coordination (Kapur et al., 1990; Grigoriadis et al., 2011, 2016). The results of the present study showed no significant effects of anesthesia on the number of chewing cycles and the duration of chewing sequence in young adults chewing elastic model food. However, sudden deprivation of sensory inputs from PMRs resulted in a significant increase in the jaw opening velocity, and duration of occlusal phase with a significant decrease in the duration of jaw-opening phases. Although deprivation of sensory inputs also resulted in an overall decrease in the EMG activity this decrease was not evident at the beginning of the masticatory sequence. On comparing the time-varying activation profile during the jaw closing phase the participants demonstrated a clear biphasic excitatory muscle drive in the control condition. However, this biphasic muscle drive was diminished and virtually absent in the anesthetized condition. Further, the difference in time-varying activation profile showed that PMRs contribute to approximately 20% of the EMG activity during the jaw closing phase. A previous study suggested that muscle spindle from the jaws is responsible for the facilitatory responses during the jaw closing phase in animals (Komuro et al., 2001). Another animal study suggested that periodontal afferents are responsible for the quick buildup of masticatory forces, but other afferents (e.g., muscle spindles) contribute to the hardness-dependent change of masticatory forces, especially during cortically induced rhythmic jaw movements (Hidaka et al., 1997). It is further suggested that low-threshold somatosensory receptors in skin, mucosa, periodontium, temporomandibular joint, etc. and their afferent inputs to the central nervous system are also suggested to contribute to the jaw muscle activity and jaw movement (Trulsson, 2006). However, our results indicate that a fraction (i.e., 20%) of the EMG activity during the jaw closing phase is also contributed by the PMRs.

### Chewing Sequence

Studies have suggested that face motor cortex plays a strategic role in most aspects of chewing and swallowing. Further, the face somatosensory cortex appears to guide these behaviors, but has a more limited role in chewing and swallowing (Sessle, 2006; Avivi-Arber et al., 2011). Dellow and Lund (1971) have demonstrated that the basic pattern/rhythm of mastication can be generated even in decelerate paralyzed animals. Under normal conditions, the sensory inputs to generate the basic rhythm of jaw opening and jaw closing are not conditionally required. However, sensory inputs are essential to adapt or to fine-tune the masticatory movements and forces according to the properties of the masticated food, or to compensate for the sudden perturbations (Lund, 1991). Animals deprived of afferent inputs can still chew, but their chewing movements appear clumsy (Morimoto et al., 1989). In agreement with these findings, our results showed that there were no significant effects of anesthesia on the number of chewing cycles and duration of the chewing sequence and the rhythm of masticatory movements. While, on the one hand, studies in humans have shown uncompromised chewing rhythm due to food hardness or with advancements in age (Horio and Kawamura, 1989; Bishop et al., 1990; Peyron et al., 2002). On the other hand, studies have also shown a decrease in chewing rhythm with an increase in tooth loss (Slagter et al., 1993) or altered masticatory pattern. However, in the current study we observed that chewing can occur without optimum sensory input and the rhythm of the chewing sequence is not compromised by a sudden deprivation of sensory inputs due to local anesthesia. These results are similar to the findings of the previous studies where there was no significant difference in the number of chewing cycles and the duration of the chewing sequence between natural dentate and people with bimaxillary implant prosthesis (Grigoriadis et al., 2011).

### Jaw Kinematics

In the current study, sudden deprivation of sensory inputs did not alter the vertical and lateral jaw movement amplitudes but caused an increase in jaw opening velocity, duration of occlusal phase with a significant decrease in duration of jaw-opening phase. These findings are contrary to the observations in patients with implant-supported prosthesis who demonstrate smaller lateral displacement of the mandible during the first chewing cycle (Grigoriadis et al., 2015). It was suggested that the “chopping-like” movement of the mandible observed in patients with implant or tooth supported prosthesis are to facilitate proper positioning of the food on the dental arch. In the process, the prosthesis patients tend to have a narrower, shortened dental arch in comparison to the natural dentate (Grigoriadis et al., 2015). However, there was no change in the amplitude of the lateral jaw movement after sudden deprivation of sensory information in the current study. This result could imply that narrower jaw movement is perhaps a learnt/adjusted behavior and a compensatory mechanism in dental prosthesis users. It is suggested that this behavior is exhibited to counter/minimize food escape from between the teeth, during chewing, compared to the momentaneous loss of sensory inputs in the current study. Previously, it has been shown that more number of food morsels escaped from between the teeth in dental prosthesis users than natural dentate controls (Grigoriadis et al., 2016). The somatosensory awareness due to the somatic sensations arising from the oral cavity provides information about the state and structure of the oral cavity along with the objects in the oral cavity (Haggard and de Boer, 2014). Lack of such information leads to decrease in somatosensory awareness and hence increase in the food escape. Further, it is also suggested that the slower jaw opening and longer duration of the occlusal phase in the current study could be due to the lack of appropriate sensory information from the PMRs during the tooth–food contact.

### Jaw Muscle Activity

Electromyographic recordings from the jaw muscles are good indicators for studying masticatory sequence patterns and movement strategies used to chew different food (Veyrune et al., 2007). The adaptation of the jaw movements to the properties of the food requires that the central nervous system have sufficient information related to forces acting on the teeth, the position and movements of the jaws, and the current state of the jaw muscles. Although several different types of mechanoreceptors in the orofacial tissues may contribute to this information (Trulsson and Johansson, 2002), the muscle spindles in the jaw closing muscles and the PMRs are considered as prime contributors (Trulsson, 2006; Turker et al., 2006; Kumar et al., 2017). Strong pressures generated during the jaw-closing phase of mastication cause the jaw closing motor neurons to fire at a higher frequency leading to accentuated jaw muscle activity. However, animal studies have shown that reduced sensory inputs from either PMRs or muscle afferents result in decrease in the accentuated jaw muscle activity required in response to increased food hardness (Lavigne et al., 1987; Morimoto et al., 1989). It was suggested that PMRs may be responsible for the sensory inputs regarding the initial tooth food contact especially at the beginning of the chewing sequence (Grigoriadis et al., 2011, 2014; Grigoriadis and Trulsson, 2018).

A strong relationship between food hardness and jaw muscle activity has been observed in humans chewing on elastic model food with controlled hardness (Peyron et al., 2002; Veyrune et al., 2007; Grigoriadis et al., 2014). The jaw muscle activity and the jaw movements adapt to the changing properties of the food during the masticatory sequence. Progression of a chewing sequence is typically characterized by an initial overall increase with a gradual decrease in the jaw muscle activity, as the chewing sequence progresses. Similarly, in the current study, we observed a higher EMG activity during the beginning of the chewing sequence and a significant decrease in the EMG activity as the food was crushed. This gradual decrease in EMG activity was evident in both the anesthetized and control conditions. Previous studies have shown that PMRs provide vital information to the jaw closing motoneurons during the beginning of the chewing sequence (Grigoriadis et al., 2011; Grigoriadis and Trulsson, 2018). However, the current study results show no significant interactions in the EMG activity between the condition and segments. This result implies that sudden deprivation of sensory inputs does not affect the EMG activity especially at the beginning of the chewing sequence.

### Temporal Profile of the Jaw Muscle Activation

It was suggested that recruitment patterns of different motor units and activation dynamics greatly influence the temporal profile and magnitude of muscle force development in a muscle (Lee et al., 2011). As mentioned above (see the section “Results”) the temporal profile showed greater EMG activity during the jaw-closing phase than the occlusal phase in the control condition. However, this difference in EMG activity between the phases was absent during anesthesia due to the sudden deprivation of sensory inputs from the PMRs. This finding suggests that a major fraction of the EMG activity in the control condition is used to overcome the resistance and crush food during the jaw-closing phase. While in the absence of sensory inputs from the PMRs the ability to increase the jaw muscle activity during the jaw closing phase is compromised. As a result, the participants were also no longer able to produce the characteristic biphasic increase in the excitatory masseter muscle drive during the anesthetized condition. In other words, they failed to generate a distinct augmentation of the jaw muscle drive during the tooth food contact at the beginning of the masticatory sequence. The lack of excitatory muscle drive in the anesthetized condition suggests that the inputs from the PMRs are critical in overcoming the resistance provided by the food during the initial tooth food contact. In the current study, the PMRs contributed to approximately 20% of the EMG activity during the jaw closing phase which was evident in the relative changes of the EMG activation between both the conditions. Therefore, lack of sensory information during the jaw closing phase compromises the regulation of masticatory forces responsible for boosting the power to overcome the resistance of the food during chewing in accordance with the animal studies (Lavigne et al., 1987; Inoue et al., 1989). These findings are similar to the observations in dental implant-supported prosthesis users where the distinct biphasic muscle drive was clearly absent in the beginning of the masticatory sequence (Grigoriadis and Trulsson, 2018). Further, previous studies have also suggested that the participants with reduced/altered sensory inputs fail to regulate the bite forces according to the specifics of the task (Kumar et al., 2017). Therefore, it is hypothesized that people with dental implants to a large degree behave like people with natural teeth with anesthesia in accordance with the previous findings (Trulsson and Johansson, 1996).

Methodological limitations are quite apparent in human experimental and clinical studies. One such limitations in the current study was the absence of an “actual” control group where normal saline would have been injected instead of the local anesthetic solution thus mimicking the exact mechanical stimulation of the needle and the pressure of flow of the fluid in the interstitial tissue during the control condition. Further, the unequal distribution of men and women among the study participants and relatively small sample size may also be a methodological concerns in these studies. Future studies should be directed at including an actual control group with equal number of men and women participants and with a relatively larger sample size in a randomized controlled study design. However, paired design where each participants was his/her own control and the normalization of EMG parameters in order to allow for cross comparison between the conditions would be positive attributes of the current study.

## Conclusion

In conclusion, sudden deprivation of sensory inputs from the PMRs affects the jaw kinematics but causes no changes in the number of chewing cycles or duration of chewing sequence. Despite the absence of changes in the EMG activity of the masseter muscle, time-varying activation profile showed absence of biphasic excitatory muscle drive in the anesthetized condition. Further, the time-varying activation profile of the masseter muscle showed that PMRs contribute to approximately 20% of the EMG activity during the jaw closing phase. Hence, sensory inputs from PMRs are responsible for the discrepancy in the activation profile of the masseter muscles during the beginning of the chewing sequence.

## Data Availability Statement

The datasets generated for this study are available on request to the corresponding author.

## Ethics Statement

The studies involving human participants were reviewed and approved by the Regional Ethical Review Board in Stockholm. The patients/participants provided their written informed consent to participate in this study.

## Author Contributions

AG performed the experiments, analyzed the data, made the figures, and prepared the first draft of the manuscript. AK analyzed the data, made the table, and drafted and edited the manuscript. MÅ analyzed the data and edited the final draft. MT conceived and designed the study, helped in data analysis, and edited several versions of the manuscript. All authors have substantially contributed and approved the final version of the manuscript.

## Conflict of Interest

The authors declare that the research was conducted in the absence of any commercial or financial relationships that could be construed as a potential conflict of interest.
